# The high-conductance state enables neural sampling in networks of LIF neurons

**DOI:** 10.1186/1471-2202-16-S1-O2

**Published:** 2015-12-18

**Authors:** Mihai A Petrovici, Ilja Bytschok, Johannes Bill, Johannes Schemmel, Karlheinz Meier

**Affiliations:** 1 Kirchhoff-Institute for Physics, University of Heidelberg, Heidelberg, Germany; 2 Institute for Theoretical Computer Science, University of Graz, Graz, Austria

## 

The apparent stochasticity of in-vivo neural circuits has long been hypothesized to represent a signature of ongoing stochastic inference in the brain [1-3]. More recently, a theoretical framework for neural sampling has been proposed, which explains how sample-based inference can be performed by networks of spiking neurons [4,5]. One particular requirement of this approach is that the membrane potential of these neurons satisfies the so-called neural computability condition (NCC), which in turn leads to a logistic neural response function.

Analytical approaches to calculating this function have been the subject of many theoretical studies. In order to make the problem tractable, particular assumptions regarding the neural or synaptic parameters are usually made [6,7]. However, biologically significant activity regimes exist which are not covered by these approaches: Under strong synaptic bombardment, as is often the case in cortex, the neuron is shifted into a high-conductance state (HCS), which is characterized by a small membrane time constant. In this regime, synaptic time constants and refractory periods dominate membrane dynamics.

The HCS is also particularly interesting from a functional point of view. In [5], we have shown that LIF neurons that are shifted into a HCS by background synaptic bombardment can attain the correct firing statistics to sample from well-defined probability distributions (i.e., satisfy the NCC). In order to calculate the response function of neurons in this regime, we are required to consider a new approach.

The core idea of this approach is to separately consider two different "modes" of spiking dynamics: burst spiking and transient quiescence, in which the neuron does not spike for longer periods. For the bursting mode, we explicitly take into consideration the autocorrelation of the membrane potential before and after refractoriness by propagating the PDF of the effective membrane potential from spike to spike within a burst. For the membrane potential evolution between bursts, we consider an Ornstein-Uhlenbeck approximation. We find that our theoretical prediction of the neural response function closely matches simulation data. Moreover, in the HCS scenario, we show that the neural response function becomes symmetric and can be well approximated by a logistic function, thereby providing the correct dynamics in order to perform neural sampling. Such stochastic firing units can then be used to sample from arbitrary probability distributions over binary random variables [4,5,8,9]. We hereby provide not only a normative framework for Bayesian inference in cortex, but also powerful applications of low-power, accelerated neuromorphic systems to highly relevant machine learning problems.
